# A simple and economical method for improving whole genome alignment

**DOI:** 10.1186/s12864-017-3734-2

**Published:** 2017-05-24

**Authors:** Huijun Mai, Tak-Wah Lam, Hing-Fung Ting

**Affiliations:** Computer Science Department, University of Hong Kong, Pokfulam road, Hong Kong, China

**Keywords:** Whole genome aligment, Seed-and-extend heuristic, Sensitivity

## Abstract

**Background:**

The recent advancement of whole genome alignment software has made it possible to align two genomes very efficiently and with only a small sacrifice in sensitivity. Yet it becomes very slow if the extra sensitivity is needed. This paper proposes a simple but effective method to improve the sensitivity of existing whole-genome alignment software without paying much extra running time.

**Results and conclusions:**

We have applied our method to a popular whole genome alignment tool LAST, and we called the resulting tool LASTM. Experimental results showed that LASTM could find more high quality alignments with a little extra running time. For example, when comparing human and mouse genomes, to produce the similar number of alignments with similar average length and similarity, LASTM was about three times faster than LAST. We conclude that our method can be used to improve the sensitivity, and the extra time it takes is small, and thus it is worthwhile to be implemented in existing tools.

## Background

Because of the recent advances in sequencing technology and genome assembly software, it is now feasible to sequence the whole genome of many species in nature. To elicit useful information from these multi-gigabase long sequences of As, Cs, Gs and Ts, one common approach is to make connections by comparing them to each other. Whole genome alignment is the computational problem of finding similar regions between two different genomes.

Current genome alignment software tools are able to align two genomes very efficiently and with only a small sacrifice in sensitivity [1–9]. Yet, it becomes very slow if the extra sensitivity is needed. This paper proposes a simple but effective method to improve the sensitivity of existing whole-genome alignment software without paying much extra running time.

## Methods

The input of the whole genome alignment problem is two multi-gigabase long DNA sequences *A*[1..*n*] and *B*[1..*m*], and it asks for finding those pairs of subsequences *A*[*i*..*j*] and *B*[*k*..*ℓ*] such that *A*[*i*..*j*] and *B*[*k*..*ℓ*] are similar. We use the alignment of *A*[*i*..*j*] and *B*[*k*..*ℓ*] to measure their similarity. To get an alignment of *A*[*i*..*j*] and *B*[*k*..*ℓ*], we insert spaces into the two subsequences to make their length equal, and put one subsequence on top of the other. We assign a score to each column of the alignment depending on whether the characters in that column are match, mismatch, or any of them is space, and the score of an alignment is the sum of the scores assigned to its columns. The similarity of *A*[*i*..*j*] and *B*[*k*..*ℓ*] is measured by the highest score given by their best alignment. Our task is to find all subsequences of *A* and *B* whose alignment scores are no less than some score threshold.

### Seed-and-extend

Most existing software tools for whole genome alignment use the "seed-and-extend" heuristic [5]. To explain this heuristic, we first note that a simple approach to solve the problem for the whole genomes *A*[1..*n*] and *B*[1..*m*] is to use dynamic programming to find, for each positions 1≤*i*≤*n* and 1≤*j*≤*m*, whether there is an alignment around *A*[*i*] and *B*[*j*] with score no less than the score threshold. Obviously, this approach is too slow. The idea of the “seed-and-extend” heuristic is to use an efficient method to find a set of promising position pairs such that for those not in this set, it is very likely that there are no similar regions around them. Then, it uses dynamic programming to find, for each promising position pair, whether there is an alignment around them with high enough score. Below, we briefly describe the basic steps of “seed-and-extend”.

(i) Index building: We distinguish the two input sequences such that one is the reference sequence and the other is the query sequence. We first build an indexing structure such as suffix array, hash table or BWT [10] for the reference sequence, and the index will enable us to find efficiently, for every short subsequence of the query, all the matching subsequences in the reference.
(ii) Seeds finding: Use the index to find all the short matching subsequence pairs of *A* and *B*. These matching pairs, which we called *seeds*, suggest a much smaller set of positions from which we find the promising positions. (Note that there are some other seeding methods such as spaced-seeds [8], adaptive seeds [5], and subset seeds [11], but in essence, they all use the index to find efficiently short matching subsequences with good scores.)
(iii) Gapless extension: The set of seeds found in the previous step suggests a much smaller set of positions for us to find similar regions. However, they are still too large for us to handle each of them using dynamic programming. This step further reduces its size as follows: for each seed, we extend it at both ends by repeatedly adding the next characters in both sequences to the alignment. Note that we will not introduce any gap in the extension, and thus this step is very fast. During the extension process, we will remember the maximum score seen so far, and if the current score is much lower than this maximum score, it means that we have gone too far, and we stop and return the alignment with the maximum score seen so far.
  To reduce the number of promising positions, we examine the set of gapless alignments returned by this step, and discard those whose score are smaller than some pre-defined threshold *D*.
(iv) Gap extension: For each of the remaining gapless alignment, we use dynamic programming to find whether there is an alignment around its starting positions with high enough scores.

### The problem of choosing the threshold *D*

It is obvious that the threshold *D* has critical effect on the efficiency and sensitivity: a low threshold allows more promising position pairs to be checked, and thus it is likely to find more similar regions, but we will also waste more time in checking more gapless alignments that do not lead to any similar regions. To demonstrate this effect, we have used the popular whole-genome alignment tool LAST [5] to align the human and mouse genome with three different thresholds, namely the default threshold *D*, and two smaller thresholds 0.9*D* and 0.85*D*. For example, as shown in the first sub-table of Table 1, by reducing the threshold from *D* to 0.85*D*, LAST can find 144,000 more alignments, but the time required is more than tripled.

**Table 1:** Comparison of LAST and LASTM

Human vs mouse				
Alignment method	Time (hours)	No. of alignments	Ave length	Ave similarity
LAST with D	3.04	2,521,923	842	61.29%
LAST with 0.9D	6.19	2,616,550	837	61.03%
LAST with 0.85D	11.85	2,665,216	834	60.95%
Our method	3.72	2,642,403	838	60.99%
Human vs dog				
Alignment method	Time (hours)	No. of alignments	Ave length	Ave similarity
LAST with D	3.18	4,533,937	905	62.99%
LAST with 0.9D	6.65	4,640,443	898	62.85%
LAST with 0.85D	12.30	4,640,877	895	62.78%
Our method	3.87	4,657,968	899	62.84%
Human vs cat				
Alignment method	Time (hours)	No. of alignments	Ave length	Ave similarity
LAST with D	4.36	4,431,635	908	62.62%
LAST with 0.9D	7.84	4,526,245	901	62.51%
LAST with 0.85D	13.26	4,571,706	899	62.46%
Our method	5.29	4,541,363	902	62.51%
Human vs cow				
Alignment method	Time (hours)	No. of alignments	Ave length	Ave similarity
LAST with D	4.59	6,437,907	785	61.65%
LAST with 0.9D	7.83	6,724,264	775	61.47%
LAST with 0.85D	12.78	6,875,343	770	61.38%
Our method	5.34	6,770,810	778	61.44%
Human vs rat				
Alignment method	Time (hours)	No. of alignments	Ave length	Ave similarity
LAST with D	4.16	8,055,476	505	62.29%
LAST with 0.9D	7.78	8,563,474	504	62.07%
LAST with 0.85D	13.27	8,836,291	503	61.96%
Our method	4.86	8,595,871	509	62.07%

Both LAST and LASTM use the HOXD70 [4] scoring scheme, and their gap existence and extension penalties are -400 and -30 respectively. Their minimum score of gapped alignments is 4500. For LAST, its default minimum score of gapless alignments is *D*=962, and for LASTM, it is 674 (≈0.7*D*). The distance *d* for the filtering step of LASTM is 2000

### Filtering

This paper proposes a simple method for increasing the sensitivity of whole genome alignment without paying much extra processing time. To justify our method, we have applied it to improve LAST, and our results show that it can increase the number of LAST’s reported alignments to the one that LAST gets with threshold 0.85*D*, but using time similar to that LAST uses with threshold *D*.

The idea of our method is to use a lower threshold, say 0.7*D* and thus we will have a larger number of gapless alignments. However, we will not pass all of them to the gap extension step. Those with score no less than *D* will be passed as usual. But for those with score between 0.7*D* and *D*, we do not have enough confidence in them, and they need to go through another test first.

**Fig. 1:**
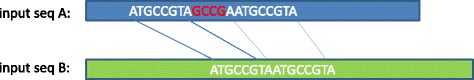
A pair of similar regions with insertion

We use Fig. 1 to explain the motivation of our test. The figure shows a pair of similar regions. Note that if we remove the insertion *GCCG* in the middle of the input sequence *A*, the two subsequences will be identical and will give a gapless extension with high score. However, the insertion breaks the upper sequence and the gapless extension step would stop at the first eight characters *ATGCCGTA*. Thus, unless we set a low enough threshold, this gapless alignment will be discarded without further examination, which is not correct because it is easy for dynamic programming to remove the insertion *GCCG* to find the similar regions. However, our example has some property that other useless gapless alignments miss; there is another gapless alignment (the one with the nine characters *AATGCCGTA*) follows closely. Hence, two close gapless alignments suggests the possibility that they may come from the same similar regions, and they should be passed to the gap extension step for further checking.

We now explain how to make use of this idea to increase the sensitivity without much extra processing time. Let *A* be the query sequence and *B* be the reference sequence. For ease of discussion, we use the notation (*A*[*i*..*j*],*B*[*k*..*ℓ*]) to denote the best alignment of the two subsequences *A*[*i*..*j*] and *B*[*k*..*ℓ*]. We use LAST as an example. We will insert the following filtering step between LAST’s gapless extension step and gap extension step, and run LAST using a lower threshold *ε* *D*, where 0<*ε*<1 and *D* is LAST’s default threshold:

Let *S* be the set of gapless alignments with score between *ε* *D* and *D*. For each alignment (*A*[*i*_*o*_..*j*_*o*_],*B*[*k*_*o*_..*ℓ*_*o*_])∈*S*, we will remove it from *S* if (i) there is no following gapless alignment within a distance of *d* from it (i.e., there is no alignment (*A*[*i*..*j*],*B*[*k*..*ℓ*]) following with *i*−*j*_*o*_≤*d* and *k*−*ℓ*_*o*_≤*d*), and (ii) there is no preceding alignment within a distance of *d* from it (i.e., there is no alignment (*A*[*i*..*j*],*B*[*k*..*ℓ*]) preceding with *i*_*o*_−*j*≤*d* and *k*_*o*_−*ℓ*≤*d*).

Then, those gapless alignments with threshold no less than *D*, together with those still in *S* will be passed to the gap extension step.

Note that the set of gapless alignments found by our method is not smaller than that found by LAST with threshold *D*, and our method will not introduce any error because all gapless alignment will be further checked by the gap extension step. As shown below, the filtering step of our method is efficient and easy to implement.

- Step 1:  Select the set *S*_*o*_ of gapless alignments with score greater than or equal to *D*, and select the set *S* of gapless alignments with score between *ε* *D* and *D*.
- Step 2:  Radix sort the gapless alignments (*A*[*i*..*j*],*B*[*k*..*ℓ*])∈*S*_*o*_∪*S* in ascending order of *i*, the starting position of *A*[*i*..*j*].
- Step 3:  Scanned the sorted list, and for each alignment (*A*[*i*..*j*],*B*[*k*..*ℓ*])∈*S* scanned, check if there is any gapless alignment *S*_*o*_∪*S* following in the list within distance *d*, and if yes, move it to *S*_*o*_. (Note that the checking for (*A*[*i*..*j*],*B*[*k*..*ℓ*]) can stop as soon as we reach an alignment (*A*[*i* ^′^..*j* ^′^],*B*[*k* ^′^..*ℓ* ^′^]) with *i* ^′^>*j*+*d*.)
- Step 4:  Radix sort the gapless alignments (*A*[*i*..*j*],*B*[*k*..*ℓ*])∈*S*_*o*_∪*S* in descending order of *j*, the ending position of *A*[*i*..*j*].
- Step 5:  Scanned the sorted list, and for each alignment (*A*[*i*..*j*],*B*[*k*..*ℓ*])∈*S* scanned, check if there is any gapless alignment *S*_*o*_∪*S* following in the list within distance *d*, and if yes, move it to *S*_*o*_.
- Step 6:  Pass *S*_*o*_ to the gap extension step.

## Results and discussion

To justify our method, we have modified LAST as described in the previous section, and used it to align the human genome (≈ 3*G*) to the genome of mouse (≈2.8*G*), of dog (≈2.4*G*), of cat (≈2.5*G*), of cow (≈2.7*G*), and of rat (≈2.8*G*). We use the human genome as the reference, and LAST will build an index for it. These genomes were all downloaded from [12].

Our experiments are run on the Intel Core i7-3930K 3.2 GHz processor, and we use 6 cores with 12 threads. For verification of our results, our program can be downloaded via the link https://github.com/Maihj/LASTM.

To evaluate the sensitivity of LAST (version 588) and our modification LASTM, we compared the number of alignments produced by each software and measured their quality based on the length and similarity. We suppose that the more alignments with similar quality produced, the higher the sensitivity.

Table 1 compares LAST’s performance (with different threshold on the minimum score of gapless alignments) with that of our method with threshold is set to 0.7*D*, and the distance *d* for the filtering step is set to 2000. The table shows that our method runs in time similar to that of LAST with threshold *D*, but the number of alignments reported is significantly larger, which is close to that reported by LAST with threshold 0.85*D*. Also, the table shows that the quality of the alignments returned by our method is similar to that returned by LAST; they have similar average length, and similar average similarity (i.e., the percentage of identical columns over the length of the alignments).

Furthermore, Table 2 showed the number of reported alignments that fall in some gene regions downloaded from [12] (i.e., at least 50% of each of the subsequences in the alignment overlaps with some gene regions of the corresponding input sequences). We find that our method has similar increase in output alignments when we focus on gene regions.

**Table 2:** No. of reported alignments falling in some gene regions

Alignment method	Mouse	Dog	Cat	Cow	Rat
LAST with *D*	1,078,259	72,651	10,854	1,154,227	1,557,117
LAST with 0.9*D*	1,107,882	73,829	11,017	1,187,743	1,636,078
LAST with 0.85*D*	1,122,373	74,470	11,101	1,205,915	1,678,187
Our method	1,119,804	74,005	11,024	1,196,821	1,647,855

## Conclusions

This paper proposes a method to increase the sensitivity of whole genome alignment tools that use the "seed-and-extend" heuristic. Our method is simple and easy to implement, and the extra time it takes is small, and thus it is worthwhile to be implemented in existing tools.
